# Precise Coulometric Titrations of Potassium Dichromate

**DOI:** 10.6028/jres.067A.047

**Published:** 1963-10-01

**Authors:** George Marinenko, John K. Taylor

## Abstract

A method has been developed for the precise assay of potassium dichromate by constant-current coulometric titration with ferrous ions generated at a platinum cathode. It, is shown that one-half gram samples of the dichromate can be titrated with a standard deviation of 0.003 percent.

## 1. Introduction

This paper reports results of a continuing investigation to evaluate coulometric methods of analysis and to develop highly precise and accurate analytical methods for important materials, based upon this technique. Earlier papers described the coulometric titrations of acids and bases [1] 1 and of halides [2]. The present research is concerned with a typical oxidation-reduction titration, the analysis of potassium dichromate.

The application of constant-current coulometric titrations to the analysis of dichromate is not novel [3, 4, 5], but highly precise and accurate results have not been reported. The method developed in the present investigation consists of the reduction of dichromate with electrogenerated ferrous ion. The technique and procedure have been refined to permit results reliable to a few parts in 100,000, which are about two orders of magnitude better than those reported by previous investigators.

## 2. General Considerations

The conditions for 100-percent efficient generation of ferrous ion at a platinum cathode are readily obtained from current density versus potential studies of the supporting electrolyte alone and with several additions of ferric ion. This technique has been employed by Lingane and co-workers for the evaluation of the current efficiency of generation of ceric ion [6] and of titanous ion [7].

**Figure 1:**
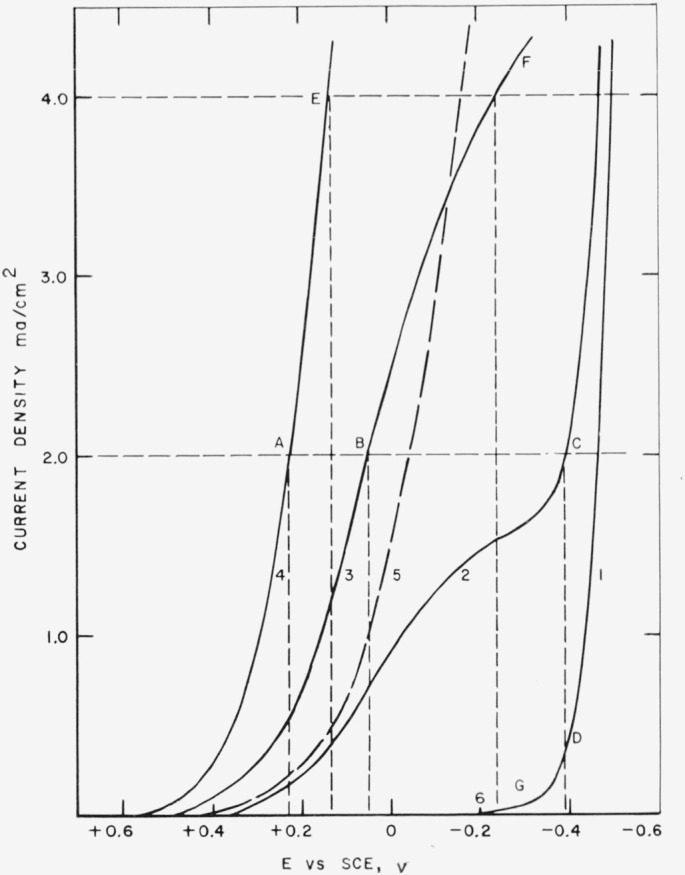
Current density, potential curves for the reduction of ferric ion at platinum cathode. 2 *M* H_2_SO_4_2 *M* H_2_SO_4_; 0.01 *M* Fe^+++^2 *M* H_2_SO_4_; 0.03 *M* Fe^+++^2 *M* H_2_SO_4_; 0.2 *M* Fe^+++^2 *M* H_2_SO_4_; 0.2 *M* Fe^+++^; 1 *M* H_3_PO_4_

The results of a study of the reduction of ferric ion at a platinum cathode are shown in figure 1. Such curves are readily and quickly obtained by the use of an *X–Y* recorder in which the potential of the platinum electrode with reference to a saturated calomel electrode is recorded on the *X*-axis while the voltage drop across a standard resistor in series with the electrolysis cell is recorded on the *Y*-axis. The latter values are related to current density from knowledge of the value of the resistor and the area of the electrode used. By manually varying the potential applied to the electrolysis cell, the curves relating current density to electrode potential are quickly obtained.

In figure 1, curve 1 represents the data obtained for the supporting electrolyte, 2 *M* sulfuric acid. Curves 2, 3, and 4 represent the data obtained on successive additions of ferric ion to the acid. The estimation of the current-efficiency for the generation of ferrous ion is illustrated by the following examples.

Consider the solution represented by curve 2. At a current density of 2 ma/cm^2^, the potential of the platinum electrode was found to be about −0.39 v (point C). At the same potential, the current density for the electrode immersed in the supporting electrolyte alone was 0.3 ma/cm^2^. The fraction of current producing ferrous ions is evidently (2.0−0.3)/2.0 or 0.85 corresponding to a current efficiency of 85 percent. For the same current density, the projections of points B and A from curves 3 and 4, respectively, on curve 1 indicate no current due to reduction of the supporting electrolyte and a resulting current efficiency of reduction of ferric ion of essentially 100 percent.

At a current density of 4 ma/cm^2^, solution 3 was reduced at a potential of about −0.24 v (point F) and this potential was obtained at a current density of 0.05 ma/cm^2^ for the supporting electrolyte alone (point G). The efficiency of reduction of solution 3 under this condition would be (4.0−0.05)/4.0 = 0.99. The projection of point E indicates a negligible current due to reduction of the supporting electrolyte and 100 percent efficiency for reduction of solution 4.

**Figure 2:**
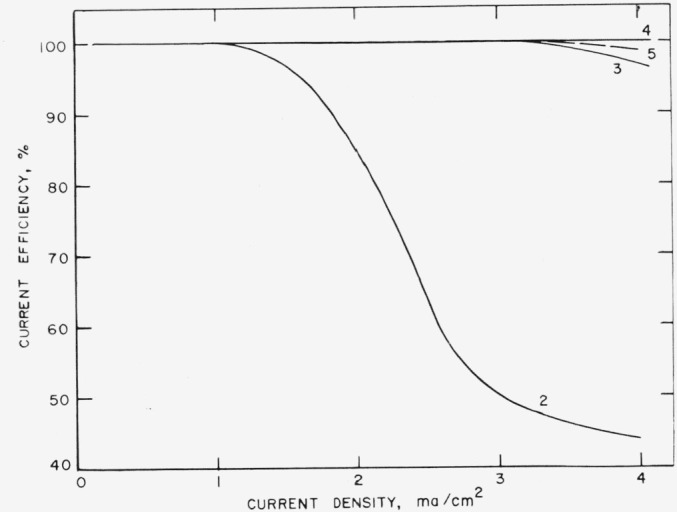
Current density, efficiency diagram for electrogeneration of ferrous ion at platinum cathode. 2. 2M H_2_SO_4_; 0.01 M Fe^+++^3. 2M H_2_SO_4_; 0.03 M Fe^+++^4. 2M H_2_SO_4_; 0.2 M Fe^+++^5. 2M H_2_SO_4_; 0.2 M Fe^+++^; 1 M H_3_PO_4_

From calculations like those described above, the curves shown in figure 2 were plotted. It is evident that solutions more concentrated than 0.01 *M* with respect to ferric ion in a supporting electrolyte of 2 *M* sulfuric acid can be reduced with a current efficiency of 100 percent, provided the current density is less than 1.2 ma/cm^2^.

It is also indicated in figure 1 that the presence of phosphoric acid is not essential for theoretically efficient reduction of ferric ion to ferrous ion, as has been reported by previous investigation [3]. In fact, it is evident that presence of phosphoric acid lowers the limiting current-density for efficient reduction of ferric ion.

The conditions selected for this work consisted in the pregeneration of ferrous ion from a solution 0.4 *M* with respect to ferric ion, in a supporting electrolyte of 2 *M* sulfuric acid, at current densities never exceeding 0.6 ma/cm^2^. The pregeneration technique, described later, reduced the ferric ion concentration to 0.3 *M* but this was well above the limit for efficient generation of ferrous ion.

**Figure 3:**
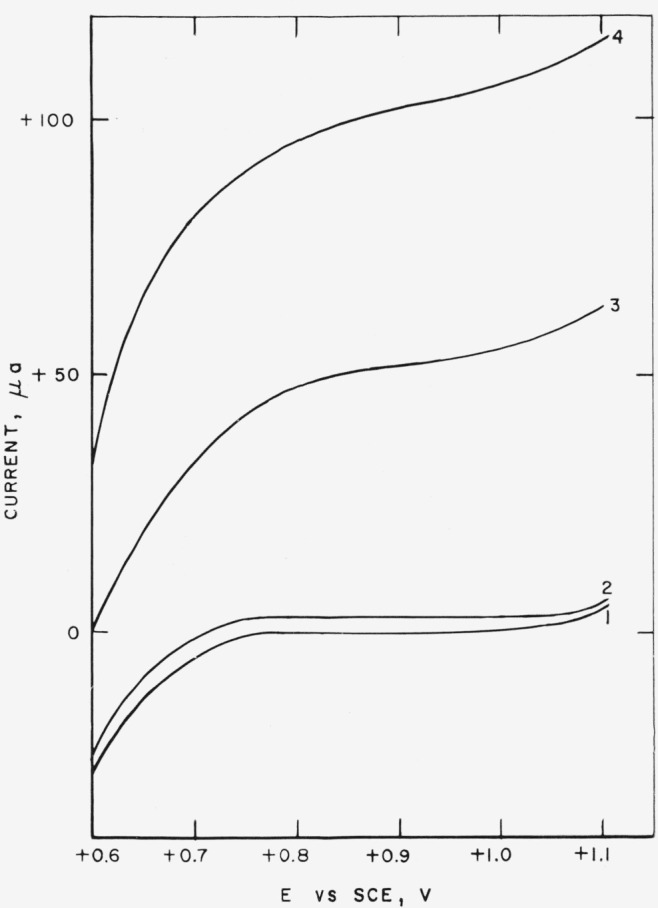
Current, potential curves for oxidation of ferrous ion at a polarized platinum electrode. 2*M* H_2_SO_4_; 0.5 *M* Fe^+++^; 40 *μ*eq K_2_Cr_2_O_7_2*M* H_2_SO_4_; 0.5 *M* Fe^+++^; 40 *μ*eq Cr^+++^; 0.5 *μ*eq Fe^++^2*M* H_2_SO_4_; 0.5 *M* Fe^+++^; 40 *μ*eq Cr^+++^; 10 *μ*eq Fe^++^2*M H*_2_SO_4_; 0.5 *M* Fe^+++^; 40 *μ*eq Cr^+++^; 20 *μ*eq Fe^++^

The end points of these titrations were determined amperometrically using a polarized platinum indicator anode and a saturated calomel electrode as the nonpolarizable reference cathode. Using this electrode system the current-voltage curves shown in figure 3 were obtained. An applied-potential of + 0.85 v is midway on the diffusion-current plateau for the reaction Fe^++^→Fe^+++^+*e*^−^. In actuality, this plateau is not completely flat; however, this voltage corresponds to a region where the indicator current is the least dependent on the applied voltage.

From studies of Anson [8, 9], it is evident that the oxide film which is formed on platinum affects the behavior of the ferrous-ferric couple at the electrode. According to him, the reduction of such a film is easily accomplished by ferrous ion in a sulfuric acid medium. If such a film were not removed prior to generation of ferrous ion, it would cause positive errors in the final analysis of oxidant, dichromate in the present case. Taking precautions to avoid such interferences, the electrodes (both the indicator anode and the generator cathode) were pretreated by immersion for 10 min in a chromic acid solution with subsequent rinsing, followed by a 10 min immersion in 0.1 *M* ferrous ammonium sulfate solution in 2 *M* H_2_SO_4_. Thus the electrode surfaces were freshly “reactivated” prior to each titration.

All calculations were based on the 1961 C^12^ atomic weight scale [10]. From it the molecular weight of K_2_Cr_2_O_7_, which was used, was calculated to he 294.191_8_.

The value of the taraday constant which was used in the calculations was 96,487.2 coul/g-equiv (based on the determination of D. N. Craig et al. [11] and corrected to the C^12^ atomic weight scale).

## 3. Apparatus and Procedure

### 3.1. Apparatus

The titration cell used in this work was similar to the one described in a previous communication [2]. The anode and cathode compartments are separated by a connecting tube containing a series of porous glass frits to provide two intermediate sections. Tubes sealed to the tops of these sections permit application of suction or pressure to fill or empty the compartments. A silicic acid gel plug, prepared by gelation of sodium silicate solution with sulfuric acid, was formed on the fine-porosity disk of the anode compartment to eliminate flow of electrolyte. The plug remained for long periods of time without degeneration provided it was kept wet with supporting electroly te.

A piece of corrugated platinum foil (5 cm × 16 cm) served as the generator cathode. The anode was constructed from sheet lead (12 cm × 10 cm × 0.2 cm) which was contoured to fit the anode compartment of the cell.

The electrical circuit was the same as used previously [1, 2]. The current was adjusted manually to maintain the IR drop across a standard resistor constant and equal to the voltage of a saturated standard cell. Because the dummy resistance only approximated that of the electrolytic cell, a momentary initial imbalance was invariably observed. Such imbalances were small and corrected quickly so that they could not affect the titrations by more than 0.00001 percent.

The saturated standard cell and the standard resistor used in this work were calibrated by the appropriate sections of the Bureau. Although the resistor was placed in a thermostated oil-bath there was always a slight temperature rise of the resistor due to heat dissipation. The correction for this temperature change was applied using the mean value of the resistor for a given titration and the temperature coefficient for manganin.

The time was measured by means of a 10 kc-quartz crystal controlled time interval meter (TIM) whose calibration was checked with respect to standard frequency signals of WWV; the calibration was found to be accurate to at least 1 ppm.

Near the equivalence point of the titration, it was more convenient to pass current increment-wise using a commercially-available constant-current couometric source. The current and timing accuracy of this apparatus were calibrated and the *It* product was found to be accurate to ±0.005 percent. Since the fraction of the total titration performed with this instrument was of the order of 0.0005, the errors encountered as a result of the use of this equipment were negligible.

The amperometric indicator system consisted of a platinum foil electrode (1 cm^2^ in area) and a saturated calomel electrode with a 3-percent agar-agar gel in 0.1 *N* potassium chloride in a salt bridge. A recording polarograph was used as the source of applied emf (0.85 v) between these two electrodes and also as the microammeter for measuring or recording the indicator current.

All weighings were done on a 20 g capacity microbalance of the single-arm, constant-load type and were precise to ±0.003 mg. All weighings were corrected for air buoyancy.

### 3.2. Procedure

The material used in this work was potassium dichromate NBS standard sample 136b. Visual examination revealed an occasional dark-colored crystal which may or may not contribute a certain amount of inhomogeneity to the sample. The material was dried at 110 °C for 24 hr and cooled in a desiccator for a period of about 4 hr before weighing.

One-half gram samples contained in a platinum boat were weighed by the method of substitution using a calibrated 500 mg tantalum weight. Sample weights agreed with the nominal value and with each other within 3 mg. The difference between the mass of the sample and the calibrated mass was determined on the optical scale of the balance. The weighed samples were transferred from the boat into 30-ml weighing bottles and stored in a desiccator until used.

The weighings were done in sets, each representing a number of samples weighed on the same day. Measurements were made of air temperature, barometric pressure, and relative humidity during the weighings and the appropriate corrections for air bouyancy were applied.

The supporting electrolyte was prepared in advance and was 0.4 *M* with respect to ferric ion and 2 *M* with respect to sulfuric acid. Ferric ammonium sulfate, reagent grade salt, or electrolytically oxidized ferrous ammonium sulfate served as the source of the ferric ion.

The anode and cathode compartments were filled with 100 ml of 2 *M* sulfuric acid and 120 ml of supporting electrolyte, respectively. At this point, about 1 ml of 0.005 *N* potassium dichromate was delivered into the cathode compartment to remove traces of ferrous ion and to facilitate the pretitration step described later.

Dissolved air was removed from the catholyte by purging with nitrogen gas that was introduced through the coarse frit of the side compartment adjacent to the cathode compartment. The nitrogen used for this purpose was pretreated by passing it through a heated copper-filled tube to remove oxygen and successively through towers containing acidified potassium permanganate, ferrous ammonium sulfate, and supporting electroly te, respectively.

After purging, the catholyte was permitted to flow into the intermediate compartments to the extent that it would just cover the bottom of each compartment, thus establishing electrolytic contact between the cathode and anode compartments. This was accomplished by application of suction to each intermediate compartment.

The catholyte was then pretitrated by passage of increments of current equivalent to 0.200 *μ*eq, using the 0.643 ma current range of the coulometric power supply. At the conclusion of each increment, the indicator current was observed. This was small and essentially constant up to the equivalence point, exhibited a curvature in the vicinity of the equivalence point, and became a linear function of the ferrous ion concentration with slope of about 5.0 *μ*a/*μ*eq beyond the equivalence point. This linear portion was extrapolated graphically and its intersection with the zero-current line was taken as the end point.

After completion of the pretitration, the intermediate compartments were rinsed by repeated emptying and filling with catholyte by applying suction or nitrogen pressure as required. The walls of the cathode compartment and its cover were rinsed by withdrawing catholyte using a syringe in which a glass tip replaced the hypodermic needle. The tip was bent so that the expelled catholyte would wash down all parts of the cathode chamber. The final reading of the indicator current was then taken from which the amount of over-titration of the pretitration step was determined.

The intermediate compartments of the cell were then filled with catholyte and ferrous ion was electrogenerated at constant current using the high precision circuit and the TIM. The amount of ferrous ion generated was a predetermined quantity sufficient to reduce about 99.95 percent of the dichromate in a given sample.

After the generation of this major portion of ferrous ion, a dry funnel was inserted into the cover of the cathode compartment through an auxiliary opening which was stoppered with a piece of glass rod during the time of generation of ferrous ion. The stem of this funnel reached slightly below the level of the liquid in the cell. The weighing bottle containing the weighed sample was inverted into the funnel and lightly tapped, so that all visible crystals of the dichromate were delivered into the cell. A complete dissolution of sample took place in about 5 min after delivery into catholyte. Following dissolution of sample the intermediate compartments were successively emptied by applying nitrogen pressure until only the bottoms of these compartments were wetted enough to make the electrical contact between the anolyte and the catholyte. The compartment which is adjacent to the cathode compartment was rinsed several more times, by permitting the catholyte to flow into it, and by forcing the same out with nitrogen pressure. This was done because a perceptible amount of ferrous ion diffused into that compartment during the course of its generation. Thus, it required more than one rinse to remove all ferrous ion which was entrapped in the glass frits. The second compartment contained an imperceptible amount of ferrrous ion.

**Figure 4:**
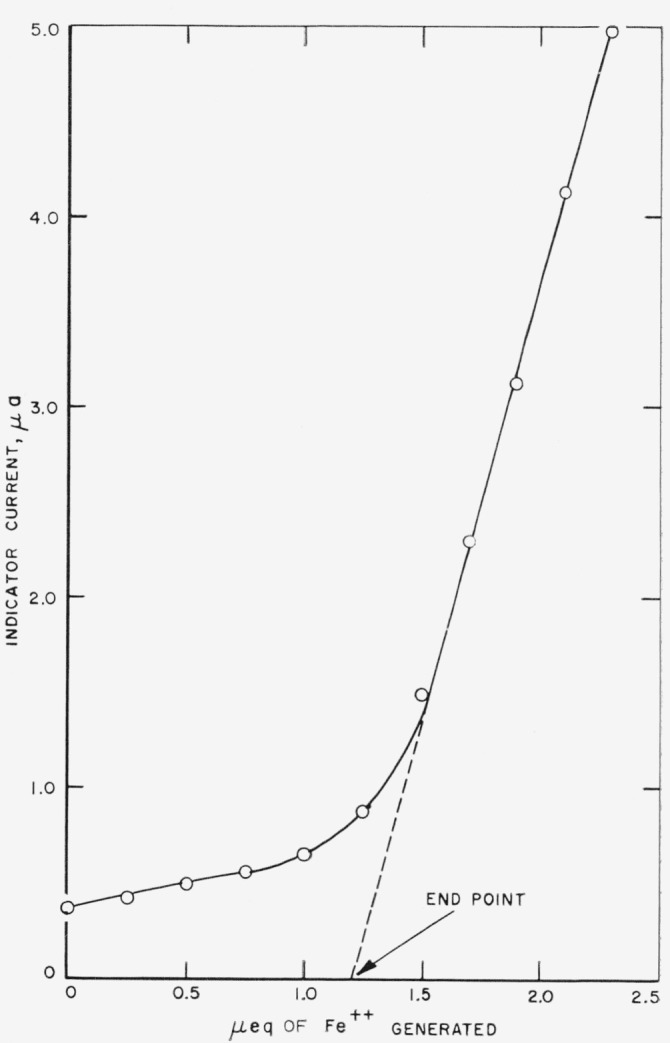
Typical end-point determination.

At this point the titration was continued increment-wise, using the coulometric current source as in the case of pretitration. When the first rise of the indicator current was observed, the: cell walls, the cell cover, and the intermediate compartments were rinsed as already described, and the weighing bottle which originally contained the sample and the delivery funnel were rinsed with catholyte withdrawn from the cell by the syringe. This procedure was repeated after each succeeding increment The extrapolation of the indicator current line, where it becomes a straight line function of the ferrous ion concentration was again taken as the end point at the point where it intersects the zero current line. A typical end-point determination is shown in figure 4.

It is obvious that the amount of ferrous ion equivalent to the dichromate is the amount generated to the end point plus the excess which was generated in pretitration.

## 4. Design of Experiments

In the initial stages of this investigation it became evident that there was a definite effect associated with the history of the supporting electrolyte. Results obtained with a fresh electrolyte appeared to be slightly yet appreciably higher than when a sample was added to an electrolyte previously used for an analysis. To verify this effect and also to detect any possible systematic errors varying with time, the analytical program outlined in the following was adopted.

Samples were weighed in sets consisting of two to six members, all weighings being made within a period of an hour or two. These samples were titrated sequentially using pretreated electrolyte except that nine samples were withdrawn in a random manner for analysis in untreated electrolyte.

The pretreated electrolyte consisted of solution that had been previously used in a coulometric titration of dichromate, or one in which a number of repeated reductions and oxidations had been performed. This latter procedure was accomplished by reduction of the supporting electrolyte (in the same electrolysis cell) for about 500 sec with a 100 ma current followed by reversal of the current. This procedure was repeated several times. The platinum electrode which was used for this pretreatment was “reactivated” as already described between each current reversal. After the final oxidation there was a small amount of ferrous ion present that had not undergone electrolytic oxidation. This residual amount was oxidized by addition of dilute solution of dichromate until the diffusion current of the ferrous ion reached a very low value (e.g., 0.5 *μ*a).

**Figure 5:**
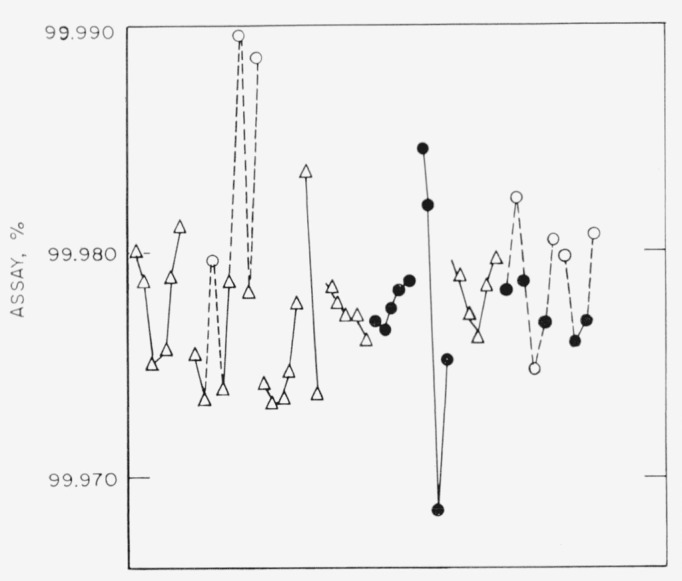
The sequence of experiments. △, previously used electrolyte○, untreated electrolyte●, electrolyte, treated by alternate reduction and oxidation

The sequence of experiments is shown in figure 5.

## 5. Results

The results for titrations of ten sets of samples in pretreated supporting electrolyte are given in columns 2 to 11 of table 1. Set XI is composed of a number of samples taken from the sets as indicated by the vacant spots labeled “c” in the table.

The average value for the assay of this material using pretreated electrolyte is 99.977_2_ percent with the standard deviation of all measurements being 0.002_9_ percent. The significantly higher value of 99.982 percent was obtained from samples titrated in the untreated supporting electrolyte. These samples also showed a higher standard deviation of 0.0049 percent.

**Figure 6:**
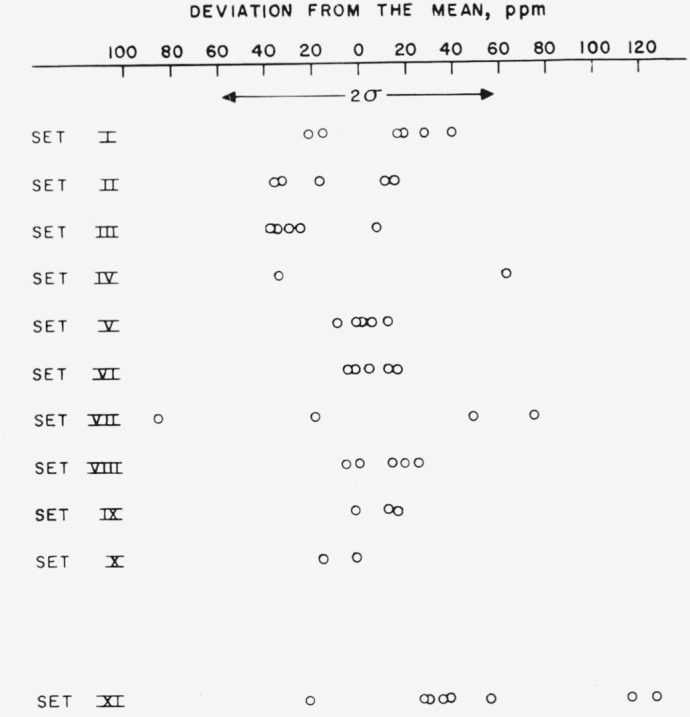
Precision of analysis. Set number XI is separated as it was not included into calculation of the mean.

The results of table 1 are plotted with respect to analytical sequence in figures 5 and 6. The results for a given series are interconnected. Dashed lines are drawn to show the time relationship for samples determined in the untreated electrolyte and emphasize that their results were not used in computing the average line nor the standard deviation limits. No significant trends with respect to time both with respect to sets and for the entire sequence are evident.

The difference between results with pretreated and untreated supporting electrolyte is obvious. Undoubtedly, some reducible impurity was present in the supporting electrolyte the kinetics of reduction of which is slow, so that it was not completely removed by the pretitration procedure. On the other hand, a longer electrolytic pretreatment and/or higher concentrations of ferrous ion had the beneficial effect in removing this impurity.

It is recommended that for titrations of potassium dichromate in which high accuracy is a serious consideration, the supporting electrolyte should be pretreated either by repeated reduction and oxidation, or by titrating a sizable sample in the electrolyte and disregarding the first result.

The precision of the method is high, in that a standard deviation of only 0.002_9_ percent, or 29 ppm, was obtained. The accuracy of the method is more difficult to evaluate in that pure reference materials are not available for calibration purposes.

The assay value, 99.977_2_ percent, is in good agreement with the certified oxidizing power, 99.98 percent, for standard sample 136b, which is given in the provisional certificate of the National Bureau of Standards dated June 30, 1961.

This certified value is based upon comparison with predecessor samples 136 and 136a, the former having been standardized with respect to pure iron and arsenious oxide. The present sample 136b was also standardized with respect to uranium metal of high purity and the provisionally certified value of 99.98± 0.01 percent represents the reliability of the value based on both analytical uncertainty and the agreement of cross-checks in which such factors as uncertainties in atomic weights are involved.

In view of the agreement of the coulometric results with other analytical data, and the fact that the conditions were such that theoretical current efficiency should have been realized, it is concluded that the accuracy of the results is consistent with the analytical uncertainty.

## 6. Discussion

This investigation emphasizes the high precision and accuracy that can be attained in constant-current coulometry. In common with the acidimetric and argentimetric titrations already reported, the determination of oxidants to a few parts in one-hundred thousand has been accomplished and uncertainties of only a few parts in a million are a definite possibility.

Coulometric methods are absolute methods in that the results depend directly upon the reliability of measured quantities. This is in contrast to many analytical methods which are comparative in principle, in that an unknown is compared with a material of known composition.

The factors affecting the precision and accuracy of coulometric titrations have been discussed in previous papers [1, 2]. In the present work, much effort was expended to minimize all errors to produce results of high reliability. An attempt has been made to evaluate the precision of all measurements involved. These are summarized in the following:

Weighings were precise to about 3 *μ*g, which amounts to 0.0006 percent in the case of 0.5 g samples and uncertainties in the buoyancy corrections did not exceed 0.0003 percent. Manual control of the current was maintained within a 0.5 cm galvanometer deflection which would amount to 0.00015 percent. Timing errors are not believed to exceed 0.0001 percent for the 10,000—second period of electrolysis. Uncertainties in the current due to temperature fluctuation of the standard resistor were no more than 0.00007 percent, while endpoint uncertainties may have amounted to about 0.02 *μ*eq which represents 0.0002 percent in a 10-meq sample. On the basis of these estimates an overall precision of 0.0008 percent should have been attained, which is about one-third of the standard deviation of the results.

There are of course other sources of error, magnitudes of which are difficult to estimate. Such indeterminate errors include mechanical losses due to transfer of sample, spray losses, and residual amounts of oxygen in the cell. Errors due to inhomogeneity of sample could be considerable since small samples (0.5 g) of incompletely purified material (99.98 percent purity) were used. Efforts were made to minimize some of the indeterminate errors by the washing procedure already described and by pretreatment of the electrolyte.

The stability of the generating current was greatly improved by the pregeneration technique, and this was the reason for its adoption. In preliminary work, it was found that the current became quite unsteady when about 30 percent of a 10 meq sample of dichromate which had been added directly to the supporting electrolyte was reduced. Potential measurements showed that the cathode potential shifted abruptly at this point from that of the dichromate-chromic couple to that of the ferric-ferrous couple. Undoubtedly, due to concentration polarization effect, the primary electrode reaction changed from the reduction of dichromate to reduction of ferric ion, well in advance of the stoichiometric end point. The transition was too rapid to adjust for by manual means and consequently large uncertainties were introduced. The pregeneration of the ferrous ion as already described eliminates this uncertainty.

Another improvement in precision of the endpoint determination resulted from the pretitration technique. Some uncertainty was introduced by small but measurable variations in the current prior to the complete reduction of dichromate (residual current). However, by alternate reduction and oxidation in the region of the end point, it became evident that extrapolations of the linear residual-current curve to zero current gave reliable values for a sample added to one previously titrated, and the pretitration technique described earlier was adopted. Also, it was hoped that this procedure would eliminate blank corrections, but it was soon discovered that a pretreatment of the supporting electrolyte was required as well.

## 7. Conclusions

This investigation has shown that potassium dichromate can be titrated coulometrically with precision and accuracy which is equal to or exceeds that obtainable by the most careful conventional methods of chemical analysis. The method has further advantage in that the reagent is generated in situ which eliminates some of the cumbersome techniques of the conventional chemical analysis. The amount of this generated reagent is measured in terms of physical quantities—current, time, and the faraday constant, all of which are more precisely and accurately measurable than the certification of conventional standard reference materials.

It has also conclusively shown that a pretreatment technique is required for supporting electrolyte if the results of highest obtainable degree of accuracy and precision are desired.

## Figures and Tables

**Table 1:** Summary of results

Assay											
Sample number	Set I a	Set II a	Set III a	Set IV a	Set V a	Set VI a	Set VII a	Set VIII a	Set IX a	Set X a	Set XI b
										
1	99.980_0_	99.975_5_	99.974_2_	99.983_5_	99.978_5_	99.976_9_	99.984_6_	99.978_9_	(c)	(c)	99.979_7_
2	99.978_8_	99.973_6_	99.973_4_	99.973_7_	99.977_7_	99.976_6_	99.982_0_	99.977_1_	99.978_3_	99.976_0_	99.989_6_
3	99.975_1_	(c)	99.973_5_	………	99.977_2_	99.977_5_	99.968_5_	99.976_4_	(c)	99.976_9_	99.988_6_
4	99.975_7_	99.973_9_	99.974_7_	………	99.977_1_	99.978_4_	99. 975_2_	99.978_5_	99.978_7_	(c)	d100.000_1_
5	99.978_9_	99.978_7_	99.977_8_	………	99.976_2_	99.978_7_	………	99.979_6_	(c)	………	99.982_6_
6	99.981_2_	(c)	………	………	………	………	………	………	99.976_9_	………	99.974_8_
7	………	99.978_3_	………	………	………	………	………	………	(c)	………	99.980_5_
8	………	(c)	………	………	………	………	………	………	………	………	99.979_8_
9	………	………	………	………	………	………	………	………	………	………	99.980_8_
Average	99.978_3_	99.976_0_	99.974_7_	99.978_6_	99.977_3_	99.977_6_	99.977_6_	99.978_1_	99.978_0_	99.976_5_	99.982_1_
Standard deviation for each set	0.002_4_	0.002_4_	0.001_8_	0.004_9_	0.000_9_	0.000_9_	0.006_0_	0.001_3_	0.001_0_	0.000_5_	0.004_9_

Overall average					99.977_2_, *σ*=0.002_9_						

a Pretreated supporting electrolyte.

b Untreated supportine electrolyte.

c Samples taken to be titrated in Set XI.

d Not included in the average.

## Footnotes

1 Figures in brackets indicate the literature references at the end of this paper.
